# The Spring Phenology of Arboreal Beetles and Spiders and Their Potential as Alternative Prey for Birds Mistimed With Peak Caterpillar Abundance

**DOI:** 10.1002/ece3.74489

**Published:** 2026-10-28

**Authors:** Ivan Bialy, Jack D. Shutt, Megan A. Stamp, Albert B. Phillimore, Kirsty H. Macphie

**Affiliations:** ^1^ Institute of Ecology and Evolution University of Edinburgh Edinburgh UK; ^2^ Department of Zoology University of Cambridge Cambridge UK

**Keywords:** alternative prey, beetles, diet‐switching, match/mismatch hypothesis, phenological distribution, spiders

## Abstract

The match/mismatch hypothesis (MMH) predicts that consumers that are asynchronous with a key ephemeral resource will have reduced fitness. However, by diet‐switching to an alternative resource with which it is synchronous, a consumer may be able to buffer the negative fitness consequences of asynchrony. The trophic interaction between spring arboreal caterpillars and insectivorous passerine consumers is a well‐established MMH study‐system, though caterpillars are not the only prey guild used. Here, we examine the phenological distribution of beetle and spider abundance, two alternative prey guilds utilised by many breeding passerines. Using data collected across a 220 km transect of Scotland, encompassing 44 mixed deciduous woodland sites, we address four questions: (i) Do beetle and spider abundance show a peaked phenological distribution during spring? (ii) Do the phenological distributions of different taxa vary among years and tree taxa? (iii) Does the overall abundance of either taxon vary among tree taxa or (iv) with temperature? We also compare the average phenological distribution of beetles and spiders to the caterpillar peak, considering whether there is potential for diet‐switching for birds mistimed with the peak in caterpillars. We found beetle abundance consistently peaked in early June, while spider abundance decreased linearly throughout spring. Overall, our results demonstrate both tree taxon‐specific and annual variation in the abundance and phenological distributions of both alternative prey guilds, with spider abundance also increasing with spring temperature. Our results demonstrate the potential for diet‐switching for mistimed birds, as the abundance of beetles was consistently higher than caterpillars throughout spring and spider abundance was higher earlier in the season, prior to the peak in caterpillars.

## Graphical abstract

The abundance and phenological distribution of alternative prey will determine whether or not they hold the potential to buffer asynchronous consumers from the negative consequences of phenological mismatch. We explore the spatiotemporal variation in spider and beetle abundance and consider their potential as alternative prey for birds mistimed with the caterpillar peak.

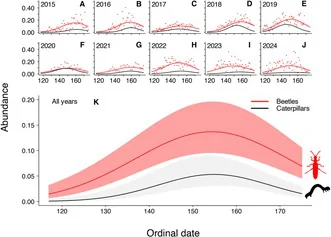

## 1 Introduction

Some of the best‐studied biological consequences of anthropogenic climate change are the shifts in the timing of life‐history events (Parmesan and Yohe 2003; Thackeray et al. 2016). At mid‐high latitudes, many consumer species have evolved to coincide periods of peak energetic demand, such as reproduction, with the arrival of more benign abiotic conditions and optimal resources in spring. When a key food resource is ephemeral, this may generate selection for consumers that are more synchronous with resource timing (Martin 1987; van Asch and Visser 2007). In warmer years and locations, spring arrives earlier and consumers can at least partially maintain synchrony with the timing of ephemeral resources through phenotypic plasticity (Visser and Both 2005; van Asch and Visser 2007), using environmental cues to signal suitable conditions. However, in a changing climate, interacting species may respond to environmental cues at different rates (Thackeray et al. 2010) or their cues may differ, leading to phenological asynchrony—a temporal mismatch between consumer demand and resource availability, as described by the match/mismatch hypothesis (MMH) (Cushing 1990). Such mismatches have the potential to be detrimental to consumer fitness and demography (Both et al. 2006; Regular et al. 2014; Miller‐Rushing et al. 2010), ecosystem functioning (Beard et al. 2019) and economically important processes like agricultural pest herbivory (Cannon 1998; Jepsen et al. 2010). Studying the drivers of variation in phenology among species is therefore increasingly important in understanding how food chains may be disrupted in a warming world.

The MMH is often studied in the context of simplified food chains that focus on individual prey species or guilds comprised of taxonomically similar species (Visser et al. 1998; Both et al. 2006; Regular et al. 2014). However, this is likely an oversimplification of more complex food webs, since many consumer species are trophic generalists that can utilise alternative prey guilds when a primary prey species or guild is unavailable (Weir and Phillimore 2024). This dietary flexibility, as observed across varied taxonomic groups, including red foxes (Baudrot et al. 2016), Atlantic cod (Jaworski and Ragnarsson 2006) and a range of passerines (Bańbura et al. 1994; Burger et al. 2012; García‐Navas and Sanz 2011; Höhn et al. 2024; Mallord et al. 2017; Samplonius et al. 2016; Wilkin et al. 2009), may serve as a critical buffering mechanism against the negative consequences of phenological mismatch.

The extent to which diet‐switching could buffer the effects of phenological asynchrony depends on the food peak architecture (Vatka et al. 2016) and how the phenological distributions of primary and alternative resources differ. If both resources share a peaked distribution of abundance, then the capacity for diet‐switching will be influenced not just by the timing of its peak, but also its height and duration (Macphie et al. 2026). When both resources entirely overlap in their phenological distribution (Figure 1A), there is no potential for diet‐switching to provide a buffer for consumers that are mismatched with their key resource. By contrast, where the alternative resource follows a broader, taller distribution (Figure 1B) or differs in timing to the primary resource (Figure 1C), then there is the potential for diet‐switching to provide a buffer.

**FIGURE 1:**
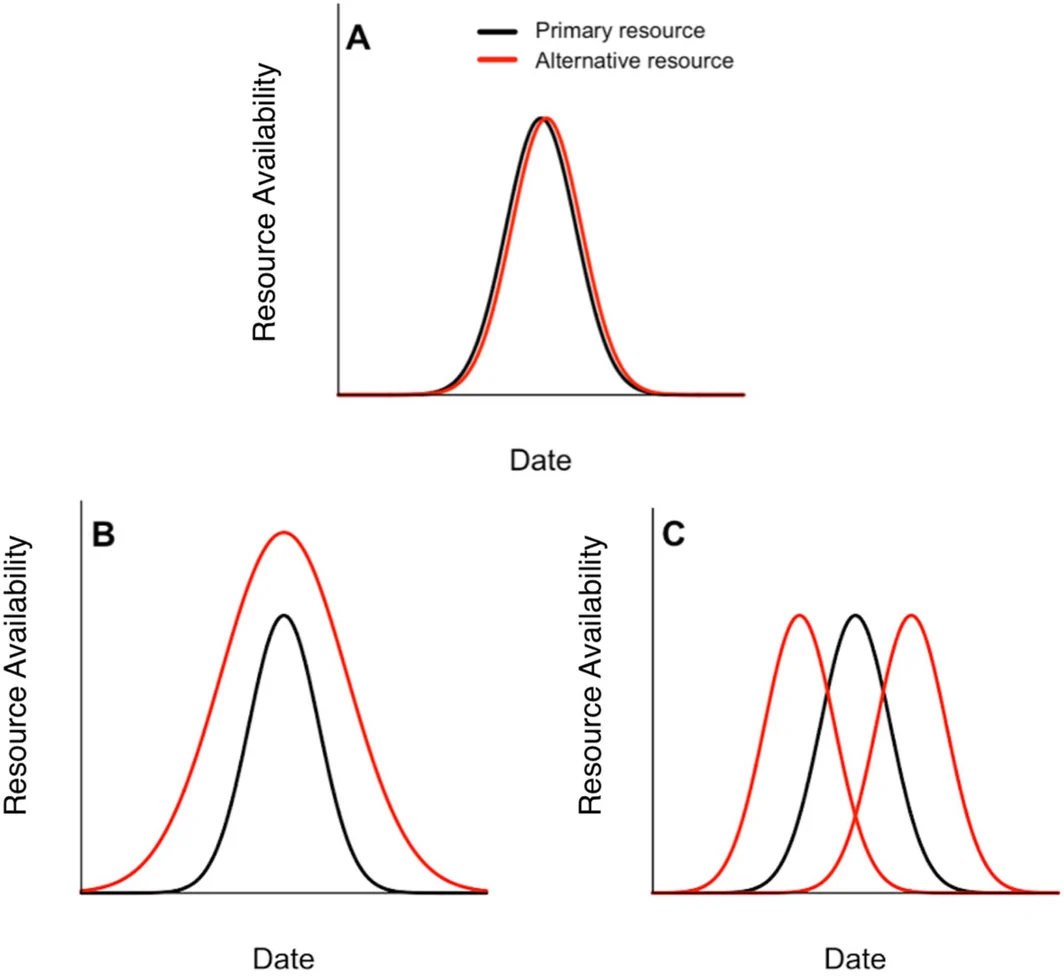
Examples of ways in which the phenological distributions of primary and alternative resources may differ. (A) Phenological peaks are very similar in timing, height and width. (B) Height and width of alternative resource peak are greater than that of primary resource. (C) Timing of primary and alternative resource peaks differ, while height and width of peaks are comparable.

A prominent system for studying phenological mismatch is the food chain linking deciduous trees, caterpillars (primarily Lepidopteran larvae) and insectivorous woodland birds (Visser et al. 1998; Both et al. 2006; Charmantier et al. 2008). The spring peak in caterpillar abundance coincides with the timing of bud burst on trees before the leaves develop chemical defences and become unpalatable (Visser et al. 1998; Feeny 1970). For many breeding bird species, caterpillars are a preferred prey guild and key resource, meaning that synchronous timing with the caterpillar peak can be important for bird productivity (Visser et al. 1998; Buse et al. 1999; Visser et al. 2005). However, these birds also rely on other prey, including spiders and beetles (Betts 1955; Höhn et al. 2024; Mallord et al. 2017; Naef‐Daenzer et al. 2000; Maziarz and Wesołowski 2010; Shutt et al. 2020) and undergo dietary shifts when caterpillars are less abundant (García‐Navas and Sanz 2010; Samplonius et al. 2016; Nicolaus et al. 2019). Therefore, the fitness consequences of asynchrony with the caterpillar peak may depend on the availability of alternative prey. In addition, this multitrophic system tends to be studied in oak‐dominated habitats (Perrins 1979; Smith et al. 2011; García‐Navas et al. 2013), while many deciduous woodlands comprise a range of tree species that are used for foraging by the birds in this system (Perrins 1979; Burger et al. 2012). As the availability of prey and their potential to contribute to the diet of insectivorous birds is likely to vary among woodland compositions, it is important to account for this habitat diversity when assessing the ecological impacts of phenological mismatch in complex, multitrophic systems.

Through the ontogeny of nestling birds there is evidence that the taxa dominating diet can change. In part, this is driven by the availability of certain prey, nutritional requirements and size constraints. For instance, younger nestlings may only be able to consume smaller prey (Slagsvold and Wiebe 2007). While the nutritional requirements of certain insectivorous passerines, like blue tits and great tits, are largely met by caterpillars, beetles also provide valuable protein and fat (Razeng and Watson 2014) and spiders have been shown to increase nestling growth, particularly when consumed during early chick development in pied flycatchers, blue tits and great tits (García‐Navas et al. 2013; Samplonius et al. 2016). The contribution of these alternative prey to nestling diets has been observed to increase in years of low caterpillar availability (Samplonius et al. 2016; Wesołowski et al. 2019; Nicolaus et al. 2019), consistent with dietary switching to beetles and spiders in response to low caterpillar abundance.

While different species of beetles and spiders will have distinct phenological distributions, guild‐level phenological distributions will reflect their availability as prey for birds throughout the breeding season. We expect both beetles and spiders to exhibit a peaked phenological distribution. In oak‐dominated woodlands, beetle species across a range of feeding guilds have been shown to exhibit a peaked phenological distribution in spring/summer, likely mirroring the high abundance of young foliage and prey (Leksono et al. 2005; Stork and Hammond 2013). Woodland spider abundance has similarly been shown to exhibit a peak in abundance between spring and summer (Turnbull 1960; Uetz 1975; Foelix 2011). The timing of peak abundance is likely to vary among years for both beetles and spiders, driven by variation in abiotic conditions and food availability for herbivorous and predatory species alike. Peak timing may also vary among tree taxa; evidence suggests that herbivorous beetles prefer younger leaves (Bale and Luff 1978; Ikonen 2002; Utsumi et al. 2009), so variation in leafing phenology may drive differences in peak timing among trees. Similarly, variation in prey phenology among trees could drive variation in the peak timing of spiders. The overall abundance of beetles and spiders is likely to vary among tree taxa, in part reflecting differences in species richness; in the UK, beetle species richness was shown to be highest on pine, oak, willow and birch (Kennedy and Southwood 1984) and spider species richness was found to be highest on oak, birch and willow (Southwood, Moran, and Kennedy 1982). Spider abundance is known to be influenced by prey availability and temperature (Uetz 1975; Foelix 2011) and for web‐building spiders, habitat structural complexity is also likely an important factor (Foelix 2011).

Spatiotemporal variation in temperature may drive variation in the abundance of individuals within beetle and spider guilds, contributing to spatial heterogeneity in prey availability for birds and potential long‐term implications under climate warming. The lifecycle of ectotherms is heavily influenced by temperature; low temperatures can slow larval development in beetles (Grafton‐Cardwell et al. 2005; Safranyik 1978), leading to lower survival rates and a reduction in population size. Consequently, low temperatures can limit the distribution of some beetle species at higher elevations (Safranyik 1978). Conversely, warmer conditions can accelerate larval development (Logan et al. 2003; Grafton‐Cardwell et al. 2005), leading to an earlier and higher peak in abundance. The developmental rate of spiders also increases with temperature (Li and Jackson 1996; Thorbek et al. 2003) and species richness was found to increase with temperature along an elevational gradient across 28 European countries (Finch et al. 2008). Taken together, higher temperatures across space and time may be associated with larger population sizes and higher species richness within beetle and spider guilds, potentially leading to greater overall prey availability for birds. However, this relationship may not always be consistent, as species differ in their responses to temperature and changes in guild composition may alter these patterns, producing variable distributions of prey guilds in space and time. Faster development may also shorten the time period over which each prey guild is available. Nonetheless, evidence suggests that warmer conditions can elevate peak prey abundance, as seen in caterpillars (Macphie et al. 2023).

In this study, we explore spatiotemporal variation in the abundance and phenological distribution of beetles and spiders, using a dataset collected over 10 years at 44 deciduous woodland sites across a 220 km transect in Scotland (Shutt 2018; Macphie et al. 2023). Within this study system, substantial variation in the phenological distribution of the caterpillar guild has already been identified across sites, tree taxa and years, as have the effects of temperature on caterpillar phenology (Shutt et al. 2019; Macphie et al. 2023, 2025). Here, we aim to describe the phenological distributions of arboreal beetles and spiders and drivers of spatiotemporal variation, in order to understand their potential as alternative prey taxa for mistimed avian consumers. Specifically, we investigate (i) whether abundance follows a peaked phenological distribution over spring and varies among years, (ii) how the phenological distribution varies across 10 common British tree taxa and how (iii) host tree taxon and (iv) temperature affect the average spring abundance of both groups. We also compare the average phenological distribution of beetles and spiders to that of the arboreal caterpillar peak to explore the potential for diet‐switching (Figure 1). Addressing these aims provides insights into the factors that affect the seasonal abundance of beetles and spiders and thus how the availability of alternative prey for avian consumers can vary temporally and spatially. Alternative prey availability holds potential to buffer the impact of phenological mismatch between woodland passerines and caterpillars, to varying extents among sites and years.

## 2 Methods

### 2.1 Study System

Data were collected from 2015 to 2024 across a 220 km transect through Scotland composed of 44 deciduous woodland sites (Figure S1A; for details see Shutt et al. 2018 and Macphie et al. 2023). Sites are located between Edinburgh (55.98° N, −3.40° E) and Dornoch (57.89° N, −4.08° E) and vary in elevation (Figure S1B) and tree taxon composition (Figure S2). The number of sites monitored varied in some years: 2015 = 35, 2016 = 37, 2020 = 22, 2021 = 37, 2022 = 43, 2023 = 22, 2024 = 22. Temperature at sites was measured hourly from mid‐February until the end of June each year using two data‐loggers, located on trees at opposite ends of each site. Dates are reported as ordinal (days since 31 December; 1 = 1 January) or calendar dates.

### 2.2 Invertebrate Sampling

Invertebrate sampling followed the branch beating methodology from Shutt et al. (2019). A clear plastic rubble sack, measuring 76 cm × 51 cm, was fully extended over the focal branch's foliage and closed at the maximum length. The branch was beaten 30 times by hand (about two beats per second) at a consistent force and rate, then the bag was carefully removed along with any dislodged invertebrates. We counted all beetles and spiders ≥ 5 mm in length and caterpillars ≥ 1 mm wide. The motivation for these size thresholds was to facilitate consistency in the field as smaller individuals are easily overlooked and because smaller prey will provide lower nutrition to consumers. The majority of these taxa that we observed were above these size thresholds, so we do not anticipate that this decision had a major influence on our results. We defined caterpillars as arboreal insect larvae that are similar in appearance to Lepidopteran larvae (Shutt et al. 2019). Previous work found the caterpillars sampled contained 93% Lepidoptera with the remaining 7% composed of Hymenoptera, Diptera and Coleoptera (Shutt et al. 2019).

At each site, focal trees were selected for branch beating to represent the local tree diversity (see Supporting Information in Macphie et al. 2025; mean number of trees per site in each year: 2015 = 6, 2016 = 6, 2017 = 14, 2018 = 14, 2019 = 15, 2020 = 14, 2021 = 14, 2022 = 14, 2023 = 14, 2024 = 14). The focal tree taxa sampled were alder *Alnus glutinosa* , ash *Fraxinus excelsior* , beech *Fagus sylvatica* , birch (*Betula* spp.), elm *Ulmus glabra* , hazel *Corylus avellana* , oak (*Quercus* spp.), rowan *Sorbus aucuparia* , sycamore *Acer pseudoplatanus* and willow (*Salix* spp.); certain trees were identified to genus level due to extensive intra‐genus hybridisation (Shutt et al. 2018). Focal trees at a site were selected at random, but conditional on the tree having at least one branch that was between 0.5 and 1.5 m above ground level and at least 1 m long—this was to ensure invertebrate sampling was possible. One suitable branch on each focal tree was tagged with a piece of string and held constant between years unless broken or dead. Focal trees at each site were divided into two groups and each group was beaten alternately at two‐day intervals, such that each branch was beaten once every 4 days, allowing for some recolonisation between beatings. Branch beating began each year at all sites when 45% of focal trees had reached the first leaf stage of their phenology (with the exception of 2020 when branch beating started on day 130, 9th May, the earliest permitted due to the COVID‐19 pandemic) and continued until the end of the field season (15–20 June). Yearly sampling dates were: 2015 = 125–175, 2016 = 130–173, 2017 = 123–167, 2018 = 129–172, 2019 = 117–168, 2020 = 130–170, 2021 = 133–175, 2022 = 119–170, 2023 = 127–171, 2024 = 121–172. The motivation to start beating when leafing was already underway was to monitor invertebrates throughout the spring while minimising damage to immature buds and leaves on trees.

### 2.3 Statistical Analysis

All analyses used generalised linear mixed models (GLMM) in the lme4 package (Bates et al. 2015) in RStudio Version 1.2.1335 (R Development Core Team 2019). All models used a Poisson error structure (log link function) and were fitted using the fast algorithm (i.e., setting nAGQ = 0). Structural random terms were included in models to account for the spatiotemporal structure and replication within the data. These were site, year, each site in each year (site‐year), each day in each year (date‐year), each day at each site in each year (date‐site‐year), tree taxon, tree ID (individual tree identity), the recorder of each beating sample and a residual (observation level) term; for descriptions of all random terms see Table S1. Prior to analysis we scaled the ordinal date variable using a z‐transformation (unscaled mean date = 147.49, SD = 14.27).

#### 2.3.1 Abundance Variance Decomposition

We decomposed the variance in the abundance of beetles and spiders, using models with either beetle or spider abundance as the response variable (Abundance models; Table S2), the intercept as the sole fixed effect and all structural random terms (as listed above), plus date as an additional random term. To assess the significance of random terms we used a likelihood ratio test to compare Abundance models with and without each random term. We expressed the latent scale variance of each variable in the Abundance models as a proportion of the total latent scale variance of the model. We also used these models to investigate whether the average spring abundance of beetles and spiders differed among tree taxa by comparing the random effect of each tree taxon. We compared the predictions of the tree taxon random effects to estimates from a model including tree taxon as a fixed effect, from which we could also attain confidence intervals for each taxon's abundance prediction (see Tree models; Table S2). As a model diagnostic, we simulated 100 datasets from each Abundance model to examine whether the Poisson GLMMs provided a suitable fit to the data and assessed whether the observed number of zeros in the abundance data fell within the predicted number. The true number of zeros fell well within the simulated predictions (Figure S3), indicating a suitable fit of the Poisson distribution to the data; this is despite 86% and 91% of branch beatings yielding zero beetles and spiders, respectively.

#### 2.3.2 Phenological Distribution of Abundance

We assessed whether beetle and spider abundance show a peaked phenological distribution using GLMMs (Beetle and Spider Peak models; Table S2), with beetle or spider abundance as the response variable and date and date^2^ as fixed effects. Here, the quadratic term allows for a peaked phenological distribution in abundance (Edwards and Crone 2021; Shutt et al. 2019). We allowed the intercept, date and date^2^ slopes to vary across years, tree taxa and sites, allowing flexibility in the shape and timing of the phenological distributions of beetle and spider abundance. We obtained year‐specific predictions of their phenological distributions by combining the intercept, date and date^2^ random effects for each year with the corresponding fixed effect. Where a peaked trend was predicted within our sampling period, we calculated the timing of the maximum abundance reached for each year. In order to compare the phenological distribution of beetles and spiders to that of caterpillars, we also ran the Peak model composition with caterpillar abundance as the response variable and calculated the phenological distribution and mean timing of caterpillar abundance in each year.

We then used the Beetle Peak and Spider Peak models to investigate how the phenological distribution of each guild varied among tree taxa. We combined the intercept, date and date^2^ random effects for each tree taxon to the corresponding fixed effect to estimate the taxon‐specific predictions. We also ran equivalent models for each tree taxon with taxon‐specific data only and removed tree taxon as a random effect (Tree‐specific models; Table S2). Including tree taxon as a random term was our preferred method. However, the fixed effect approach allowed us to consider whether the more conservative random effects approach was constraining variation in the shape of the phenological distributions compared to the fixed effects approach. Details of this analysis can be found in Supporting Information.

#### 2.3.3 Temperature Effects on Abundance

To assess the effect of temperature (from ordinal day 120—the start of branch beating—to day 163—when temperature ceased to be recorded) on beetle and spider abundance, we calculated the mean temperature for each site and year combination. We used GLMMs (Beetle and Spider Temperature models; Table S2), with beetle or spider abundance as the response variable and temperature as a fixed effect. Tree taxon was removed from the structural random terms because the prevalence of some tree taxa is correlated with spatial biogeographic gradients and temperature (Shutt et al. 2018). For comparison, we also ran the Temperature models with tree taxon as a random term; results shown in Supporting Information (Table S5).

## 3 Results

We conducted 51,319 branch beatings across 759 trees, 44 sites and 10 years. A total of 10,496 beetles and 5303 spiders were recorded, with 14% of branch beatings yielding at least one beetle and 9% yielding at least one spider. Where at least one beetle was sampled, 72% of branch beatings yielded one beetle and 17% yielded two beetles. Where at least one spider was sampled, 84% of branch beatings yielded one spider and 13% yielded two spiders. Maximum abundance of beetles and spiders per branch was 30 and 10, respectively.

### 3.1 Abundance Variance Decomposition

All terms included in the Abundance models explained variance in beetle and spider abundance (Figure 2, Table S3). The spatial random terms (site, tree taxon and tree ID) accounted for the largest non‐residual variance component for both beetle and spider abundance (28% and 32%), of which site and tree ID contributed similarly for beetles (site: 10%, tree ID: 12%) and spiders (site: 12%, tree ID: 13%), while variance among tree taxa was lower (6% and 7% for beetles and spiders, respectively). The temporal random terms (date, year and date‐year) explained 17% and 12% of the variance in beetle and spider abundance. For beetles, the highest proportion of the temporal variance was explained by date (8%), while variance in spider abundance was slightly higher among years (5%) than for other temporal terms. Beetle abundance exhibited relatively low among‐years variance (3%). The spatiotemporal category (site‐year and date‐site‐year) accounted for 15% and 13% of the variance for beetles and spiders, the majority of which was across unique date‐site‐year combinations (13% and 12%). Finally, the ‘other’ category accounted for the largest proportion of variance (44% for beetles and spiders; Figure 2), most of which was captured by the residual term (40% and 35%).

**FIGURE 2:**
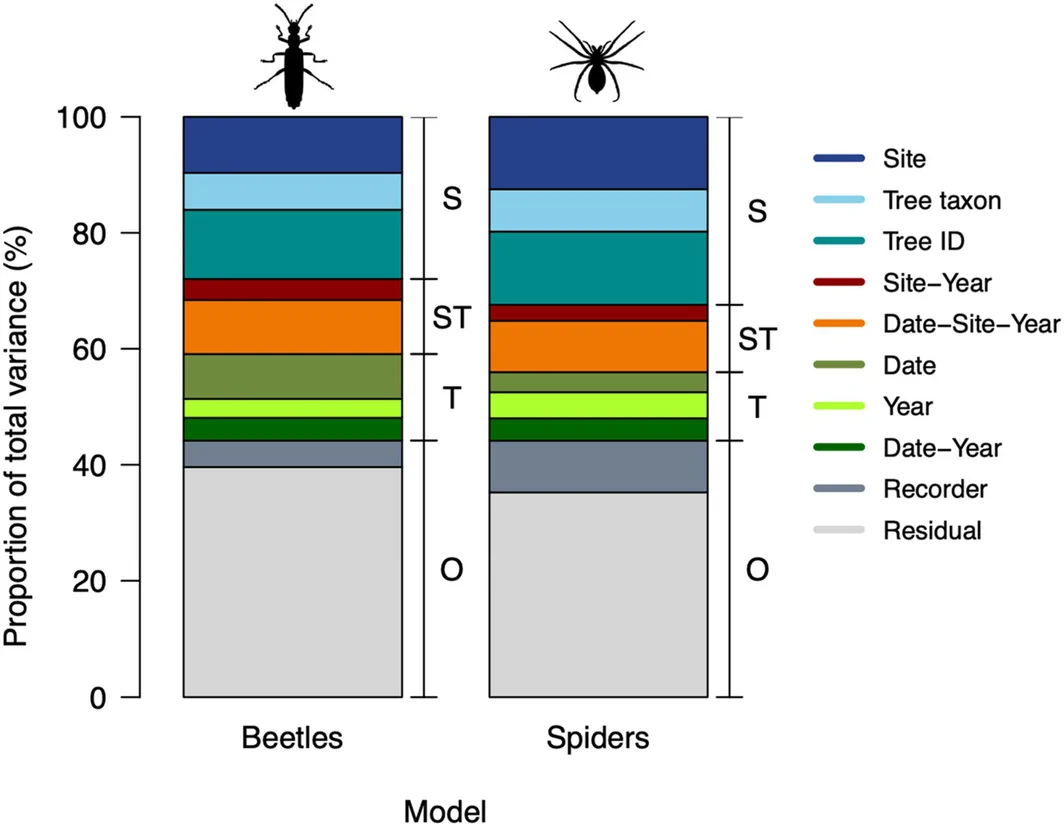
Latent scale variance components of the Abundance models for beetles and spiders as a percentage of the total variance (2.180 for beetle Abundance model, 1.592 for spider Abundance model). Vertical bars adjacent to columns show variance breakdown into four categories, spatial (S), spatiotemporal (ST), temporal (T) and other (O). Silhouettes representing each guild were sourced from PhyloPic (https://www.phylopic.org).

### 3.2 Average Spring Beetle and Spider Abundance Across Tree Taxa

Spring beetle abundance was highest on birch, followed by hazel and oak, while spring spider abundance was highest on oak, followed by hazel and elm (Figure 3). The tree taxon with the lowest abundance of both invertebrate groups was ash. Estimates from the Tree models, fitting tree taxon as a fixed effect, showed consistent patterns for beetles, with the same taxa supporting the highest and lowest abundances. For spiders, the lowest‐abundance taxon was also consistent, but the highest abundance was predicted on hazel rather than oak (Figure S7).

**FIGURE 3:**
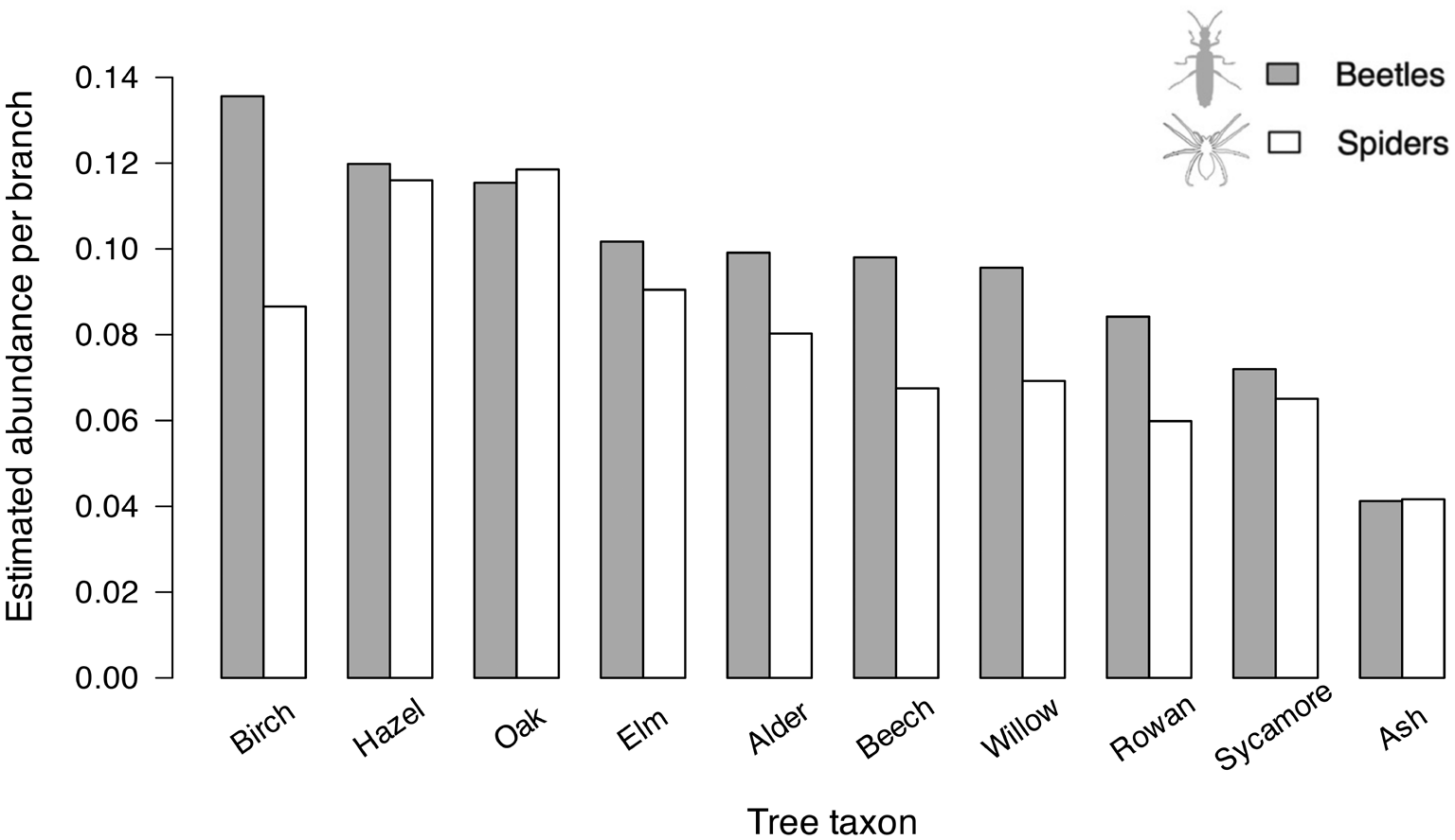
Model estimates of mean beetle (grey) and spider (white) abundance per branch for each tree taxon, calculated from the Abundance models (Table S2). Tree taxa are ordered by beetle abundance. Silhouettes representing each guild were sourced from PhyloPic (https://www.phylopic.org).

### 3.3 Phenological Distribution of Abundance

Beetle abundance showed a peaked phenological distribution throughout the spring (date^2^ slope = −0.324 ± 0.037, *p* < 0.001; Table S4, Figure 4). The intercept varied among years ($\chi_{3}^{2}$ = 76.902, *p* < 0.001) as did the date slope ($\chi_{3}^{2}$ = 75.72, *p* < 0.001), indicating the peak timing and abundance varied annually, with the highest peak occurring in 2019 (Figure 4). The date^2^ slope did not vary among years ($\chi_{3}^{2}$ = 2.704, *p* = 0.440), indicating that the shape of the phenological distribution among years was relatively consistent. The beetle and caterpillar peak dates were broadly similar across years, varying from ordinal day 149–159 for beetles and day 146–161 for caterpillars (Figure S4). The predicted peak in caterpillar abundance throughout spring falls consistently lower than the predicted beetle abundance (Figure 4K).

**FIGURE 4:**
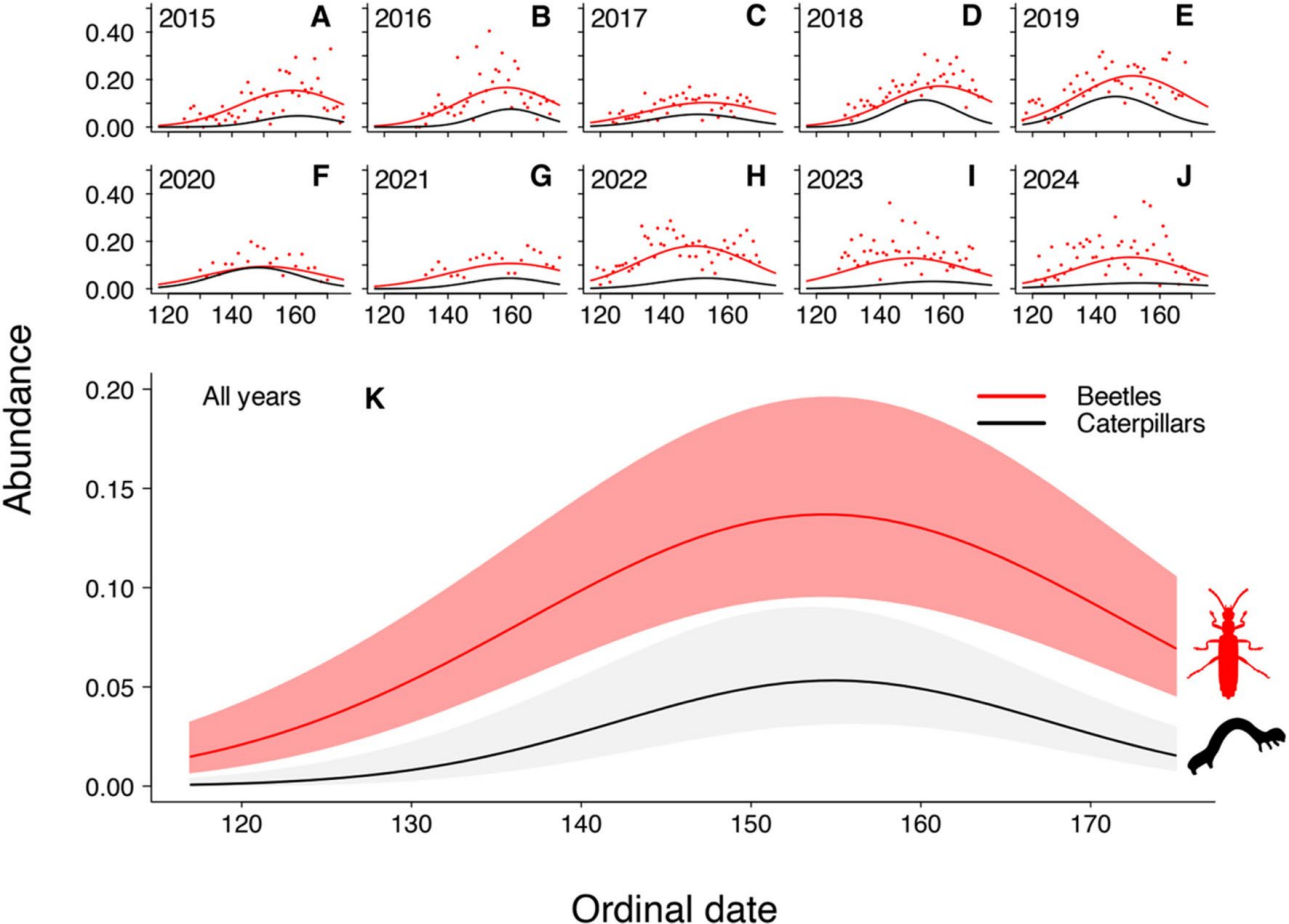
Phenological distribution of spring arboreal beetle abundance (red) per branch from 2015 to 2024 (A—J) and the average trend across all years (K), estimated from the Beetle Peak model. Plots also show the equivalent trends estimated for caterpillar abundance (black) from the Caterpillar Peak model for comparison. Individual‐year plots (A—J) show predictions of the annual trend, calculated from the fixed effects and year random effects with data points showing the predicted daily means for beetles (see Supporting Information). Plot K shows the average trend among years for both invertebrate taxa with 95% confidence intervals. Silhouettes representing each guild were sourced from PhyloPic (https://www.phylopic.org).

Spider abundance decreased throughout the spring and showed no peaked distribution (date^2^ slope = 0.025 ± 0.029, *p* = 0.375; Table S4, Figure 5). The intercept varied among years ($\chi_{3}^{2}$ = 23.678, *p* < 0.001), suggesting spider abundance differed annually, though we found no evidence of the date slope ($\chi_{3}^{2}$ = 0.402, *p* = 0.940) or date^2^ slope ($\chi_{3}^{2}$ = 0.572, *p* = 0.903) varying among years. The predicted spider abundance in early/mid spring was higher than the predicted abundance of caterpillars, with overlapping predictions from late‐May (Figure 5K).

**FIGURE 5:**
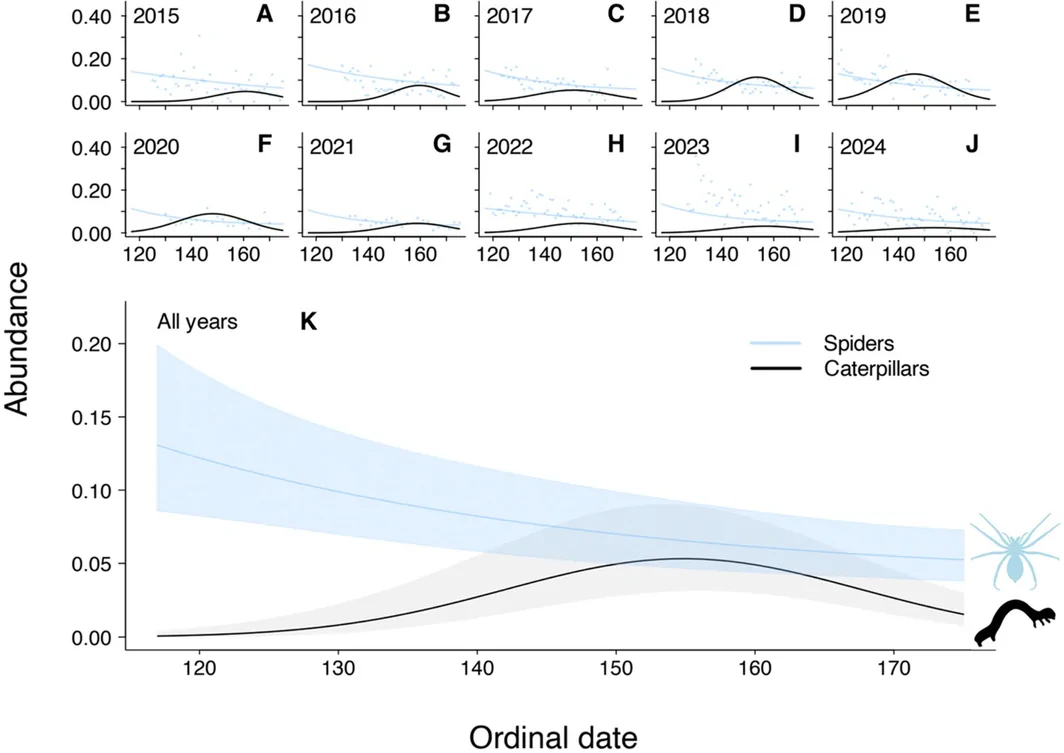
Phenological distribution of spring arboreal spider abundance (blue) per branch from 2015 to 2024 (A—J) and the average trend across all years (K), estimated from the Spider Peak model. Plots also show the equivalent trends estimated for caterpillar abundance (black) from the Caterpillar Peak model for comparison. Individual‐year plots (A—J) show predictions of the annual trend, calculated from the fixed effects and year random effects with data points showing the predicted daily means for spiders (see Supporting Information). Plot K shows the average trend among years for both invertebrate taxa with 95% confidence intervals. Silhouettes representing each guild were sourced from PhyloPic (https://www.phylopic.org).

### 3.4 Phenological Distribution of Abundance Across Tree Taxa

Models predicted a peaked phenological distribution of beetles on all tree taxa (Figure 6A). The intercept varied among trees ($\chi_{3}^{2}$ = 70.834, *p* < 0.001) as did the date slope ($\chi_{3}^{2}$ = 60.403, *p* < 0.001), indicating that the maximum abundance reached throughout spring and peak timing differed among tree taxa. The date^2^ slope did not vary among trees ($\chi_{3}^{2}$ = 4.904, *p* = 0.179), such that we have no evidence that the shape of the phenological distribution varied among taxa. Predictions derived from the random term approach (Beetle Peak model) and subset data approach (Beetle Tree‐specific models) predicted similar trends for all tree taxa (Figure S5).

**FIGURE 6:**
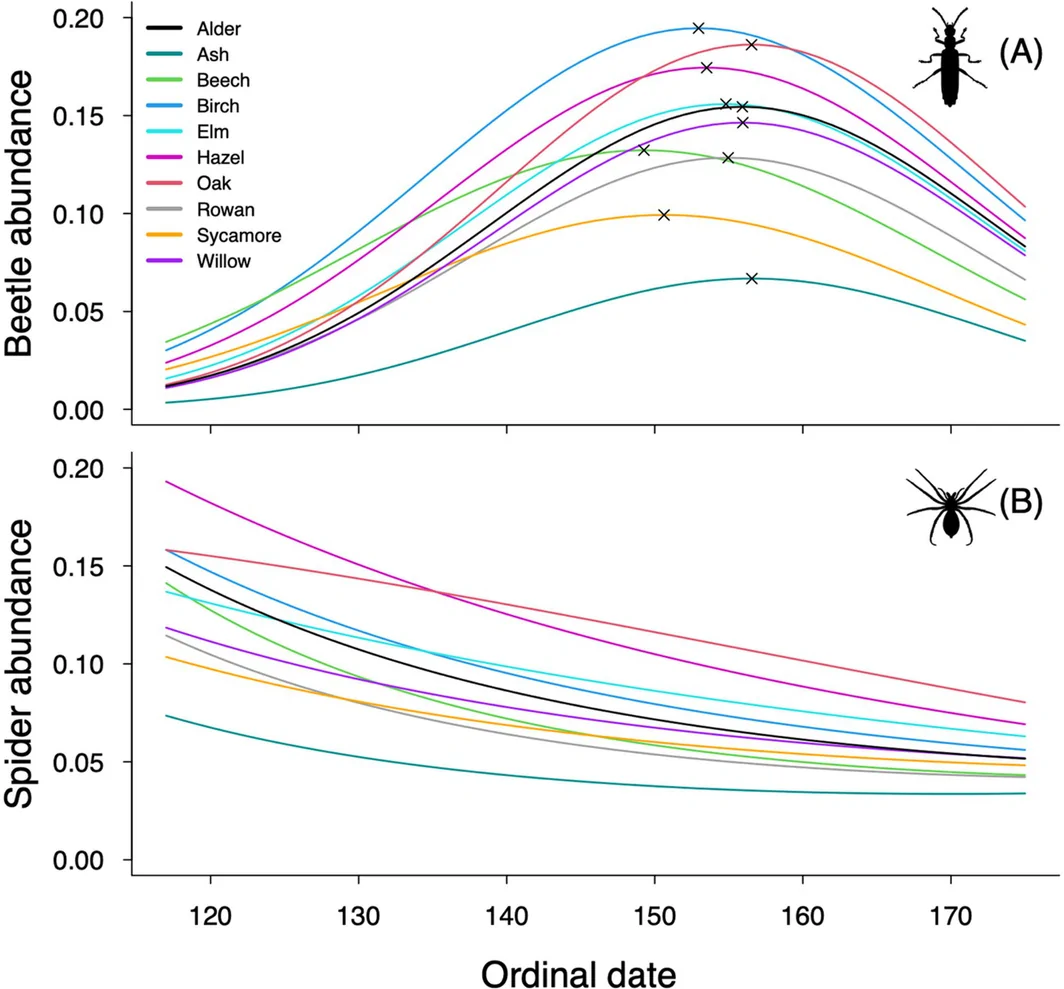
Predicted phenological distributions of beetle (A) and spider (B) abundance per branch for each tree taxon, from the Beetle Peak and Spider Peak models (see Table S2). Crosses on trend‐lines represent the predicted peak date for each tree taxon. Silhouettes representing each guild were sourced from PhyloPic (https://www.phylopic.org).

Spider abundance decreased linearly over spring on all tree taxa (Figure 6B). The intercept varied among tree taxa ($\chi_{3}^{2}$ = 66.728, *p* < 0.001); the date and date^2^ slopes did not ($\chi_{3}^{2}$ = 4.212, *p* = 0.240; $\chi_{3}^{2}$ = 2.602, *p* = 0.457), suggesting that while spider abundance varies among tree taxa, the temporal trend was relatively consistent. The main difference in tree taxon‐specific predicted trends between the random term approach (Spider Peak model) and subset data approach (Spider Tree‐specific models) was for willow, in which the Spider Tree‐specific model predicted a peaked phenological distribution (date^2^ slope = −0.380 ± 0.114, $\chi_{1}^{2}$ = 9.333, *p* < 0.01; Figure S6H), while the Spider Peak model predicted a decrease over spring (Figure 6B). For all other tree taxa, the Spider Peak model and Spider Tree‐specific models predicted similar trends (Figure S6).

### 3.5 Temperature Effects on Abundance

We found no significant effect of spring temperature on beetle abundance (Beetle Temperature model: slope = 0.017 ± 0.044, *p* = 0.701; Figure 7A), regardless of whether or not tree taxon was included as a random effect (Table S5). In comparison, for spiders we detected a positive effect of temperature on abundance (slope = 0.096 ± 0.046, *p* < 0.038; Figure 7B), corresponding to abundance increasing by 10% ^o^C^−1^.

**FIGURE 7:**
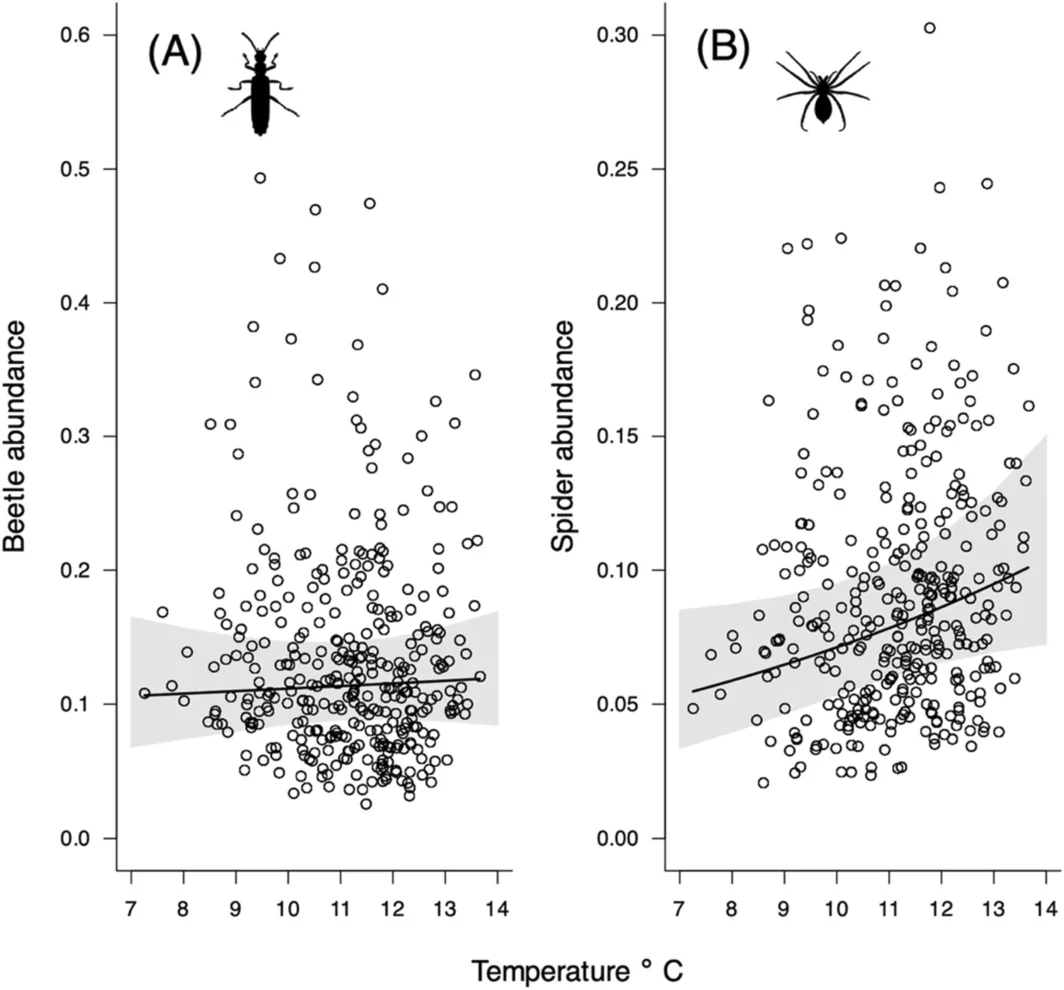
Relationships between beetle (A) and spider (B) abundance and temperature, predicted by the Beetle Temperature and Spider Temperature models (see Table S2). Data points represent the mean abundance per branch at each site in each year (see Supporting Information). Silhouettes representing each guild were sourced from PhyloPic (https://www.phylopic.org).

## 4 Discussion

Our examination of phenological and abundance patterns in spring arboreal beetles and spiders revealed that while some impacts are consistent between these two groups, other responses are strongly taxon‐specific. We found a peaked distribution of beetle abundance in spring, varying in timing and height among years and tree taxa. The average peak in beetle abundance among years was consistently higher than caterpillar abundance throughout spring (Figure 4K). Spider abundance was found to decrease linearly throughout the season and vary in height but not slope among years and tree taxa. Predicted spider abundance was higher than caterpillar abundance early in the season but remained similar after the first few weeks (Figure 5K). Overall abundance of beetles and spiders was highest on birch and oak, respectively, while ash hosted the lowest abundance of both groups, results which are comparable to the differences previously found in caterpillar abundances among trees (Macphie et al. 2025). While we found no effect of temperature on beetle abundance, the effect on spider abundance was positive.

Comparison of the average spring trends in beetle, spider and caterpillar abundance allows us to consider beetles and spiders as alternative prey for birds mistimed with the caterpillar peak, potentially buffering negative fitness consequences of asynchrony. We found that beetles were consistently higher in abundance than caterpillars, with a broader peak (Figure 4K), such that they likely provide an alternative prey for birds when caterpillars are scarce (Figure 1B). Similar beetle and caterpillar peak dates across years and tree taxa (Figures 4, 6; Macphie et al. 2025) suggest there is limited potential for buffering via distinct timing among prey guilds (Figure 1C). Spiders were predicted to be present at higher abundances than caterpillars in early/mid spring (Figure 5K), suggesting an opportunity for diet‐switching to spiders for early birds mistimed with caterpillars. Spiders have been found to make up a higher portion of pied flycatcher nestling diets prior to the caterpillar peak (Samplonius et al. 2016) and higher spider prevalence in the diet benefits nestling growth in pied flycatchers, blue tits and great tits (García‐Navas et al. 2013; Samplonius et al. 2016). These positive associations between spider prevalence in the diet and nestling growth will only be further strengthened by the relatively high abundance of spiders in the environment at a time when caterpillar numbers are low. From our model predictions, the abundance of spiders is higher than beetles early in the season, but beetle abundance exceeds that of both spiders and caterpillars after a few weeks, for the remainder of spring (Figure S8). As a result, spiders may serve as an alternative prey guild for birds that breed earlier than the caterpillar peak, whilst beetles appear more likely to be present in higher abundances for those that are late or in years where caterpillars are scarce. However, should asynchrony to the caterpillar and beetle peak increase, with birds expected to breed increasingly later than their prey, the availability of beetles will also decline making them unlikely to buffer nests that are severely delayed.

The differences observed between the phenological distributions of beetles, spiders and caterpillars suggest potential for diet‐switching within a location, but we can also consider whether these relative differences are likely to vary in space. Over a quarter of the variance in beetle and spider abundance was explained by our spatial terms, indicating the abundance of these prey guilds varies significantly across locations. High overall prey abundance may be able to compensate for trophic asynchrony (Durant et al. 2005), suggesting that locations where beetle and spider abundance is high, such as birch and oak‐dominated woodlands, may play a larger role in supporting insectivorous woodland birds at the meta‐population scale, via spatial portfolio effects (Miller‐Rushing et al. 2010; Schindler et al. 2015). However, the among‐tree taxon differences in beetle and spider abundance follow a broadly similar trend to caterpillars (Macphie et al. 2025), such that additional spatial buffering effects are unlikely to occur via diet‐switching between woodlands of different tree compositions, with the exception of alder woodlands (see below). The limited variation in peak timing among tree taxa observed in beetles and previously caterpillars (Macphie et al. 2025), is consistent with work on variation in blue tit breeding phenology that found no effect of woodland tree composition (Branston et al. 2024).

Our results support the generality of a peaked phenological distribution in beetle abundance across 10 deciduous tree taxa. This is likely driven by the emergence of young, palatable leaves and associated invertebrates, which could generate an abundance peak in both herbivorous and predatory beetles. This would be in agreement with previous phenological studies that have reported a peak in beetle pest species abundance that are correlated with crop emergence (Jeffords et al. 1983; Landolt et al. 1999) and of the beetle guild on oak, *Quercus robur* (Leksono et al. 2005; Stork and Hammond 2013). Peak timing was similar for beetles and caterpillars across years, likely due to the ephemeral food source being shared by herbivorous beetles and caterpillars. Beetle abundance was highest on birch, followed by hazel and oak, similar to trends in tree caterpillar abundance (Macphie et al. 2025). A notable distinction in relative abundance of these invertebrates is that alder hosts average to high numbers of beetles compared to other tree taxa (Figure 6), whilst it hosts very low numbers of caterpillars (Macphie et al. 2025), suggesting that beetles may be a more important prey guild for consumers breeding in alder‐dominated woodlands. While little work has looked at beetle abundance among European trees, some work has examined beetle species richness; Kennedy and Southwood (1984) found that beetle species richness was higher on native and more abundant tree taxa such as birch and oak, which may suggest beetle species richness positively correlates with guild abundance. However, where we show a high abundance of beetles on hazel, Kennedy and Southwood found it to host relatively low beetle diversity. Variance in beetle abundance and peak height among tree taxa suggests that the potential for their contribution to the diet of insectivorous passerine nestlings is liable to vary among habitats.

Variation in the beetle peak date among tree taxa could arise from interspecific variation in the spring leafing phenology of trees, though we were not able to explicitly test for differences in timing among trees. For example, one of the earlier beetle peaks was found on sycamore while the latest was on oak, which corresponds to the relative leafing phenology of these taxa (Roberts et al. 2015); yet we find that beetles peak earlier on beech than all other taxa despite the relatively late leafing phenology of beech trees. However, the narrow time difference between peak dates, occurring within 10 days of each other, may suggest the sampled beetle guild was dominated by a few generalist species, as found on birch (Southwood, Moran, and Kennedy 1982) and oak (Southwood, Mow, and Kennedy 1982; Stork and Hammond 2013), which heavily influenced the overall phenological distribution of beetles. These generalist beetle species may be locally adapted to the phenology of the most abundant tree taxon and associated prey for carnivores and feed on nearby tree taxa opportunistically, as suggested for winter moths (Tikkanen et al. 2006). To explore this idea, species inventories are necessary to establish the contributions of different species to the overall phenological distribution of beetles. Species inventories would also reveal the proportion of beetles belonging to different feeding guilds; while the majority of arboreal beetles are herbivorous, a significant proportion are predatory or xylophagous (Aikens and Buddle 2012; Stork and Hammond 2013). As the phenological distributions of different feeding guilds are known to vary due to differences in the seasonality of their food resources (Southwood et al. 2004; Stork and Hammond 2013), this may lead to a broader peak in beetle abundance than that offered by caterpillars to insectivorous birds.

Our results identified a seasonal decline in spider abundance that contradicts results from previous studies. Work exploring the phenological distribution of spiders previously found a steep increase in abundance in early spring, which plateaued before declining by mid‐summer (Turnbull 1960; Williams 1962; Cardoso et al. 2007). An initial spring peak is likely to precede our sampling period, as spiders were abundant when sampling began. The subsequent decline in abundance across the sampling period was observed in all years and on all tree taxa and may be the result of the branch beating sampling method used. Previous work used either pit‐fall traps or a comparable branch beating method, but without resampling the same branch each time (Turnbull 1960; Williams 1962; Cardoso et al. 2007). Web‐building spiders, which comprise a significant proportion of deciduous canopy spider assemblages (Aikens and Buddle 2012; Oguri et al. 2014), are highly selective about where they construct webs since silk is an energetically costly resource (Opell 1997) and are known to relocate if disturbance levels are high (Chmiel et al. 2000; Hansell 2008). Therefore, branch beating may have driven both the removal and the relocation of spiders and deterred other spiders from establishing a web on regularly beaten branches. Sampling may have caused less disturbance to spiders in previous studies, some of which sampled spiders once every 2 weeks (Russell‐Smith and Swann 1972; Abraham 1983), monthly (Williams 1962) or across different areas of focal trees each time (Turnbull 1960). Whilst the effect of our sampling method on the phenological distribution of spiders is unclear, the identified variation in abundance among years and trees continues to be of interest and relevance to avian consumers. At no point in time will the abundance of spiders be overestimated, meaning that we have confidence in the predicted higher abundance of spiders than caterpillars in early spring.

Prey abundance is an important factor in determining the spatial and temporal distribution of spiders (Halaj et al. 1998; Foelix 2011). As a result, the high spider abundance found on oak may reflect the high abundance and species richness of insects found on this genus, in comparison to other British trees (Southwood, Moran, and Kennedy 1982; Southwood, Mow, and Kennedy 1982). The high abundance of spiders and caterpillars (Macphie et al. 2025) on oak reinforces the value given to oak in describing habitat suitability for breeding tits (Wilkin et al. 2007; Bell et al. 2014), given their significant role in chick development (García‐Navas et al. 2013; Samplonius et al. 2016).

We found no effect of temperature on beetle abundance, but a positive effect of temperature on spider abundance (Figure 7). The increase in spider abundance of 10% ^o^C^−1^ is broadly consistent with previous studies and may be attributable to an increase in vegetation structure and consequent prey availability in warmer sites and years (Jiménez‐Valverde and Lobo 2007; Finch et al. 2008). This increase in abundance could reflect increasing spider species richness, higher species population sizes or a combination of the two. Beetle abundance was not correlated with temperature, contrary to previous research (McCoy 1990; Jeffords et al. 1983; Safranyik 1978). While we see no pattern in abundance at the guild level, turnover in the guild composition across the range of temperature sampled may obscure temperature effects present at the species level. Further work could also investigate whether a particular period of time better predicts beetle abundance and whether the effect of temperature on spider abundance is similar in space and time (Lovell et al. 2025).

An important consideration when interpreting our results in terms of nutrition to a consumer is that one beetle or spider may not be nutritionally equivalent to one caterpillar. Despite the high abundance of beetles, many birds appear to prefer caterpillars when available (Wilkin et al. 2009; Burger et al. 2012), suggesting higher nutritional value per unit biomass or easier digestion due to the lack of a hard exoskeleton. The nutritional value of different prey may also vary through spring, as indicated by larger chick size when spiders are fed during early chick development (García‐Navas et al. 2013; Samplonius et al. 2016). Nevertheless, the consistently higher abundance of beetles relative to caterpillars and the high early‐spring abundance of spiders, still provide alternative prey when caterpillars are scarce; for example, in alder‐dominated woodland where the caterpillar peak is low (Macphie et al. 2025) but beetles and spiders are relatively abundant (Figure 6).

Our study has focused on the *potential* for diet‐switching to spiders or beetles by examining their availability relative to caterpillars throughout spring. A valuable extension to this work would assess the availability of additional alternative prey guilds, such as Dipterans, Hemipterans and Hymenopterans (Höhn et al. 2024), to gain a fuller picture of the seasonal availability of alternative prey. In order to establish that diet‐switching occurs, one would need additional data on the items provisioned to nestlings (e.g., Samplonius et al. 2016). To go a step further and show that diet‐switching acts as a buffer, would require demonstrating that by switching to an alternative diet the fitness costs of asynchrony are reduced—testing this in this system would require additional information on the link between diet and fledging success.

In summary, the phenological distribution of beetle abundance indicates that they are likely to present an ephemeral resource for breeding insectivorous passerines in spring and occur at a similar time to caterpillars. However, while the phenology of these two taxonomic groups is similar, beetles are generally present at higher abundances than caterpillars, meaning that they may serve as an alternative resource for birds mistimed with the caterpillar peak, particularly when caterpillar peak abundance is low. The average abundance of spiders in early/mid spring sits higher than that of caterpillars, such that early birds may be buffered from the absence of caterpillars. The decreasing abundance trend through spring that we identify for spiders differs from results from previous studies, which we suggest may be a consequence of our sampling approach. The variability in beetle and spider abundance across tree taxa and sites suggests there is likely to be spatial variation in the contribution of these prey guilds to the diets of birds and their potential as alternative prey for mistimed individuals, though differences among trees largely mirror the differences observed in caterpillars. Our results reveal spatial and temporal variation in the phenological distributions and abundance of two important alternative prey guilds within the classic tri‐trophic deciduous woodland study system. Negative consequences of trophic asynchronies are of concern for a broad range of consumers at mid‐high latitudes, though research often focuses on a specific, preferred resource. Understanding the availability of alternative resources is a crucial step in determining whether diet‐switching is a viable buffering mechanism for consumers mistimed to their primary resource. Furthering our understanding of the importance of alternative prey groups for consumers will be of great value in determining the consequences of mistiming under the MMH and ongoing environmental change.

## Author Contributions

**Ivan Bialy:** conceptualization (equal), formal analysis (equal), methodology (equal), visualization (lead), writing – original draft (lead), writing – review and editing (supporting). **Jack D. Shutt:** conceptualization (supporting), data curation (supporting), funding acquisition (supporting), writing – review and editing (supporting). **Megan A. Stamp:** data curation (supporting), funding acquisition (supporting), writing – review and editing (supporting). **Albert B. Phillimore:** conceptualization (supporting), data curation (lead), formal analysis (supporting), funding acquisition (lead), methodology (equal), project administration (lead), supervision (supporting), writing – review and editing (supporting). **Kirsty H. Macphie:** conceptualization (equal), data curation (supporting), formal analysis (equal), funding acquisition (supporting), methodology (equal), supervision (lead), writing – original draft (supporting), writing – review and editing (lead).

## Funding

This work was supported by Natural Environment Research Council (NE/I020598/1O, NE/P011802/1), NERC funded PhD, NERC funded PhD through E3 DTP, NERC funded PhD through E4 DTP.

## Conflicts of Interest

There are no conflicts of interest.

## Supporting information


**Figure S1:** (A) Map of Scotland indicating the location of all 44 sites (crosses) with Edinburgh, Perth and Aviemore labelled. (B) Latitudinal elevation profile of the transect showing the locations of Perth and Aviemore.
**Figure S2:** Proportional tree species composition at each site. Sites from left to right are in order of increasing elevation (SER = 10 m.a.s.l., DNS = 433 m.a.s.l.). See Macphie et al. 2025 for habitat quantification methods.
**Figure S3:** Histogram of the frequency of observing zeros for beetles (A) or spiders (B) in data simulated under the Abundance model (100 datasets of 51,319 observations). Dashed line corresponds to the number of occasions when zero beetles or spiders were found in our dataset. Silhouettes representing each guild were sourced from PhyloPic (https://www.phylopic.org).
**Figure S4:** Comparison of the dates of peak beetle and caterpillar abundance. Peak dates were calculated from annual estimates in the Beetle Peak and Caterpillar Peak models (see Table S2); dashed line at 1:1.
**Figure S5:** Comparisons of the phenological distributions of beetles on birch (A), ash (B), oak (C), beech (D), hazel (E), alder (F), sycamore (G), willow (H), elm (I) and rowan (J), obtained using the Tree‐specific models (black) and the Beetle Peak model (red; see Table S2). Shaded areas represent 95% confidence intervals in the beetle abundance predictions from the Tree‐specific models.
**Figure S6:** Comparisons of the phenological distributions of spiders on birch (A), ash (B), oak (C), beech (D), hazel (E), alder (F), sycamore (G), willow (H), elm (I) and rowan (J), obtained using the Tree‐specific models (black) and the Spider Peak model (red; see Table S2). Shaded areas represent 95% confidence intervals in the spider abundance predictions from the Tree‐specific models.
**Figure S7:** Predicted mean and 95% confidence intervals for model estimates of beetle (black) and spider (red) abundance per branch for each tree taxon. Calculated from the Tree models (Table S2) including tree taxon as either a fixed effect (circles) or random effect (crosses). Silhouettes representing each guild were sourced from PhyloPic (https://www.phylopic.org).
**Figure S8:** Average spring phenological trend for beetles (red), spiders (blue) and caterpillars (black). Lines show the average trend among years with 95% confidence intervals.
**Table S1:** Summary of random intercept terms included throughout analyses and their utility. Random temporal (date and date^2^) slopes were also estimated across those terms highlighted with asterisks in the Peak models (see Methods—Phenological distribution of abundance).
**Table S2:** Summary of the key models used, including the response variables, fixed and random terms and the utility of each model.
**Table S3:** Latent scale variance components of the Abundance model for beetles and spiders and the significance of all variables included in analysis, determined by term deletion and model comparison using likelihood ratio tests. Total variance in Abundance models: 2.180 and 1.592 for beetles and spiders, respectively.
**Table S4:** Poisson GLMM analysing the phenological distribution of beetles and spiders over spring.
**Table S5:** Poisson GLMM analysing the effect of temperature on beetle and spider abundance per branch, including tree taxon as a random effect.

## Data Availability

All the required data and code are availablie on https://doi.org/10.5281/zenodo.23335990.
