# Supplementary material for: The Spring Phenology of Arboreal Beetles and Spiders and Their Potential as Alternative Prey for Birds Mistimed With Peak Caterpillar Abundance
Source: Ecol Evol. 2026 Oct 28;16(11):e74489. doi: 10.1002/ece3.74489 (PMC13717218; doi:10.1002/ece3.74489)
Supplement: Supplementary file 1 [file ECE3-16-e74489-s001.docx]

**Supplementary material for ‘The spring phenology of arboreal beetles and spiders and their potential as alternative prey for birds mistimed with peak caterpillar abundance’**

*Figure S1. (A) Map of Scotland indicating the location of all 44 sites (crosses) with Edinburgh, Perth and Aviemore labelled. (B) Latitudinal elevation profile of the transect showing the locations of Perth and Aviemore.*

*Figure S2. Proportional tree species composition at each site. Sites from left to right are in order of increasing elevation (SER = 10 m.a.s.l., DNS = 433 m.a.s.l.). See Macphie et al., 2025 for habitat quantification methods.*

*Table S1. Summary of random intercept terms included throughout analyses and their utility.* *Random temporal (date and date^2^) slopes were also estimated across those terms highlighted with asterisks in the Peak models (see Methods - Phenological distribution of abundance).*

| **Random term** | **Utility** |
| --- | --- |
| Site* | Allows sites to deviate in invertebrate abundance |
| Tree taxon* | Allows tree taxa to deviate in invertebrate abundance, as has been reported in other studies (Southwood et al., 1982a) |
| Tree ID | Allows for variation in invertebrate abundance among individual trees |
| Site-Year | Allows site by year combinations to deviate in invertebrate abundance on top of any site and year effects |
| Date-Site-Year | Accounts for daily fluctuations in invertebrate abundance within particular sites and years. This term should capture some effects of fine-scale spatiotemporal variation in weather. |
| Date | Allows for consistencies in temporal trends in beetle and spider abundance among years (only used in the Abundance model) |
| Year* | Accounts for annual deviations in beetle and spider abundance |
| Date-Year | Allows for variation in beetle and spider abundance on each day of each year in addition to the general date and year effects |
| Recorder | Accounts for variation in branch beating and recording technique of different recorders |
| Residual term | A numerical sequence the length of the total number of observations (30,109), accounting for overdispersion in the model (Harrison, 2014) |

*Figure S3. Histogram of the frequency of observing zeros for beetles (A) or spiders (B) in data simulated under the Abundance model (100 datasets of 51,319 observations). Dashed line corresponds to the number of occasions when zero beetles or spiders were found in our dataset. Silhouettes representing each guild were sourced from PhyloPic (*[*https://www.phylopic.org*](https://www.phylopic.org)*)*

*Table S2. Summary of the key models used, including the response variables, fixed and random terms and the utility of each model.*

| **Model** | **Response** | **Fixed effects** | **Random terms** | **Utility** |
| --- | --- | --- | --- | --- |
| Abundance model | Beetle or spider abundance | Intercept | Site, Tree taxon, Tree ID, Site-Year, Date-Site-Year, Date, Year, Date-Year, Recorder, residual term | • Test contribution of variables to total variance in beetle/spider abundance data  • Compare beetle/spider abundance across tree taxa  • Verify whether Poisson was appropriate for data |
| Peak model | Beetle, spider or caterpillar abundance | Date and Date^2^ | Date/Site*, Date/Tree taxon*, Date/Year*, Tree ID, Site-Year, Date-Site-Year, Date-Year, Recorder, residual term | • Test for a peaked phenological distribution of beetles and spiders  • Derive tree taxon and year-specific phenological distributions for beetles and spiders |
| Temperature model | Beetle or spider abundance | Temperature | Site, Tree ID, Site-Year, Date-Site-Year, Year, Date-Year, Recorder, residual term | • Test effect of temperature on beetle/spider abundance |
| Tree-specific models | Beetle or spider abundance | Date and Date^2^ | Date/Site*, Date/Year*, Tree ID, Site-Year, Date-Site-Year, Date-Year, Recorder, residual term | • Validate tree taxon-specific phenological distributions using tree taxon-specific data subsets (Fig. S5/S6) |
| Tree models | Beetle or spider abundance | Tree taxon | Site, Tree ID, Site-Year, Date-Site-Year, Year, Date-Year, Recorder, residual term | **•** Compare beetle/spider abundance across tree taxa with a measure of uncertainty (Fig. S7) |

*Date and date^2^ random slopes (see Methods)

*Table S3. Latent scale variance components of the Abundance model for beetles and spiders and the significance of all variables included in analysis, determined by term deletion and model comparison using likelihood ratio tests. Total variance in Abundance* *models: 2.180 and 1.592 for beetles and spiders, respectively.*

|  | **Beetles** | | | | **Spiders** | | | |
| --- | --- | --- | --- | --- | --- | --- | --- | --- |
| **Random effect** | **Variance** | **χ^2^** | **DF** | ***P* value** | **Variance** | **χ^2^** | **DF** | ***P* value** |
| Site | 0.211 | 120.5 | 1 | <0.001 | 0.199 | 140.92 | 1 | <0.001 |
| Tree taxon | 0.139 | 74.918 | 1 | <0.001 | 0.116 | 65.218 | 1 | <0.001 |
| Tree ID | 0.260 | 747.37 | 1 | <0.001 | 0.201 | 272.8 | 1 | <0.001 |
| Site-Year | 0.079 | 105.84 | 1 | <0.001 | 0.044 | 24.244 | 1 | <0.001 |
| Date-Site-Year | 0.203 | 101.34 | 1 | <0.001 | 0.141 | 29.099 | 1 | <0.001 |
| Date | 0.168 | 92.712 | 1 | <0.001 | 0.055 | 36.776 | 1 | <0.001 |
| Year | 0.071 | 57.626 | 1 | <0.001 | 0.072 | 33.783 | 1 | <0.001 |
| Date-Year | 0.085 | 96.451 | 1 | <0.001 | 0.062 | 39.857 | 1 | <0.001 |
| Recorder | 0.100 | 218.33 | 1 | <0.001 | 0.142 | 287.78 | 1 | <0.001 |
| Residual | 0.863 | 1414.6 | 1 | <0.001 | 0.561 | 212.49 | 1 | <0.001 |

*Table S4. Poisson GLMM analysing the phenological distribution of beetles and spiders over spring.*

| **Explanatory variable** | **Beetles** | | | | **Spiders** | | | | |
| --- | --- | --- | --- | --- | --- | --- | --- | --- | --- |
|  | **Estimate** | **SE** | ***Z* value** | ***P* value** | | **Estimate** | **SE** | ***Z* value** | ***P* value** |
| (Intercept) | -2.064 | 0.184 | -11.187 | < 0.001 | | -2.620 | 0.176 | -14.887 | < 0.001 |
| Date* | 0.312 | 0.106 | 2.946 | < 0.005 | | -0.219 | 0.039 | -5.654 | < 0.001 |
| Date^2^* | -0.324 | 0.037 | -8.769 | < 0.001 | | 0.025 | 0.029 | 0.887 | 0.375 |

*Date has been z-transformed (see Methods). Original mean date = 147.49, standard deviation = 14.27.

Random effect variances (beetles): Residual: 0.857, Date-Site-Year: 0.162, Site-Year: 0.091, Tree ID: 0.261, Site: 0.243; Date random slope across site: 0.051 (intercept - linear slope correlation = 0.10); Date^2^ random slope across site: 0.005 (intercept - quadratic slope correlation = -0.66), Recorder: 0.089, Tree taxon: 0.137; Date random slope across tree taxa: 0.026 (intercept - linear slope correlation = -0.22); Date^2^ random slope across tree taxa: 0.002 (intercept - quadratic slope correlation = 0.42), Year: 0.097; Date random slope across years: 0.066 (intercept - linear slope correlation = -0.16); Date^2^ random slope across years: 0.005 (intercept - quadratic slope correlation = -0.36).

Random effect variances (spiders): Residual: 0.562, Date-Site-Year: 0.112, Site-Year: 0.046, Tree ID: 0.202, Site: 0.181; Date random slope across site: 0.022 (intercept - linear slope correlation = -0.53); Date^2^ random slope across site: 0.004 (intercept - quadratic slope correlation = 0.04), Recorder: 0.146, Tree taxon: 0.130; Date random slope across tree taxa: 0.003 (intercept - linear slope correlation = -0.02); Date^2^ random slope across tree taxa: 0.001 (intercept - quadratic slope correlation = -0.86), Year: 0.068; Date random slope across years: 0.001 (intercept - linear slope correlation = 0.75); Date^2^ random slope across years: 0.001 (intercept - quadratic slope correlation = -0.52).

*Figure S4. Comparison of the dates of peak beetle and caterpillar abundance. Peak dates were calculated from annual estimates in the Beetle Peak and Caterpillar Peak models (see Table S2); dashed line at 1:1.*

*Figure S5. Comparisons of the phenological distributions of beetles on birch (A), ash (B), oak (C), beech (D), hazel (E), alder (F), sycamore (G), willow (H), elm (I) and rowan (J), obtained using the Tree-specific models (black) and the Beetle Peak model (red; see Table S2). Shaded areas represent 95% confidence intervals in the beetle abundance predictions from the Tree-specific models.*

*Figure S6. Comparisons of the phenological distributions of spiders on birch (A), ash (B), oak (C), beech (D), hazel (E), alder (F), sycamore (G), willow (H), elm (I) and rowan (J), obtained using the Tree-specific models (black) and the Spider Peak model (red; see Table S2). Shaded areas represent 95% confidence intervals in the spider abundance predictions from the Tree-specific models.*

*Figure S7. Predicted mean and 95% confidence intervals for model estimates of beetle (black) and spider (red) abundance per branch for each tree taxon. Calculated from the Tree models (Table S2) including tree taxon as either a fixed effect (circles) or random effect (crosses). Silhouettes representing each guild were sourced from PhyloPic (https://www.phylopic.org)*

*Figure S8. Average spring phenological trend for beetles (red), spiders (blue) and caterpillars (black) . Lines show the average trend among years with 95% confidence intervals.*

*Table S5. Poisson GLMM analysing the effect of temperature on beetle and spider abundance per branch, including tree taxon as a random effect.*

|  | **Beetles** | | | | **Spiders** | | | |
| --- | --- | --- | --- | --- | --- | --- | --- | --- |
| **Explanatory variable** | **Estimate** | **SE** | ***Z* value** | ***P* value** | **Estimate** | **SE** | ***Z* value** | ***P* value** |
| (Intercept) | -2.540 | 0.521 | -4.876 | < 0.01 | -3.631 | 0.543 | -6.687 | < 0.001 |
| Temperature | 0.022 | 0.044 | 0.496 | 0.620 | 0.092 | 0.046 | 2.008 | < 0.05 |

Random effect variances (beetles): Residual: 0.861, Site: 0.211, Tree ID: 0.260, Date-Site-Year: 0.207, Date-Year: 0.203, Site-Year: 0.079, Year: 0.049, Recorder: 0.089, Tree taxon: 0.138.

Random effect variances (spiders): Residual: 0.560, Site: 0.186, Tree ID: 0.201, Date-Site-Year: 0.141, Date-Year: 0.116, Site-Year: 0.043, Year: 0.078, Recorder: 0.140, Tree taxon: 0.116.

Tree-specific models:

We studied variation in the phenological distribution of beetles and spiders across tree taxa by comparing taxon-specific curves obtained from the Beetle Peak and Spider Peak models. These taxon-specific deviations come from a distribution around the overall trend predicted by the Peak models. To validate these predictions, we made independent models for each tree taxon (Tree-specific models; Table S2). These were fitted with beetle or spider abundance as the response variable and date and date^2^ as fixed effects. Models were only fitted with the structural random terms, described in the Methods section. We ran all 10 models (one for each tree taxon) with data for the corresponding tree taxon (Fig. S5/S6).

Obtaining data points for Peak model plots (Fig. 4, Fig. 5):

To visualise the phenological distribution of beetles and spiders in our data we plotted the predicted mean daily abundance of beetles and spiders for each year and across all years combined. These predicted means were obtained from GLMMs (one predicted mean for each day within each year) with beetle or spider abundance as the response variable and only the intercept as a fixed effect. For year-specific plots (panels A-J in Fig. 4/5), random effects were site, tree taxon and the residual term; since models were predicting daily invertebrate abundance per year, date and year were not included as random effects. Recorder was excluded as well, since the number of recorders on a single day was low which could lead to unreliable mean beetle abundance predictions, and tree ID was also excluded since, on a single day, within any particular year, any variance among individual trees would be captured by the residual term.

Obtaining data points for Temperature model plots (Fig. 7):

To visualise the effect of temperature on beetle and spider abundance, we plotted the predicted mean abundance of beetles and spiders at each site in each year, which were obtained from two GLMMs, identical to the Beetle Temperature and Spider Temperature models (Table S2) but with only the intercept as a fixed effect. The intercept coefficients for each site, year and site-year were combined to the intercept fixed effect from the corresponding Temperature model to provide the predicted mean beetle and spider abundance per site-year combination.
